# The Sri Lanka Mother and Newborn Growth (S-MaNGro) Cohort: Protocol of a Nationwide Prospective Study

**DOI:** 10.3390/mps7040061

**Published:** 2024-08-06

**Authors:** Malshani Lakshika Pathirathna, Megumi Haruna, Satoshi Sasaki, Kaori Yonezawa, Yuriko Usui, Yasuhiro Hagiwara

**Affiliations:** 1 Department of Midwifery and Women’s Health, Division of Health Sciences and Nursing, Graduate School of Medicine, The University of Tokyo, Tokyo 113-0033, Japan; mharuna@m.u-tokyo.ac.jp (M.H.); kaoriyone@m.u-tokyo.ac.jp (K.Y.); yusui@g.ecc.u-tokyo.ac.jp (Y.U.); 2 Department of Nursing, Faculty of Allied Health Sciences, University of Peradeniya, Peradeniya 20400, Sri Lanka; 3 Global Nursing Research Center, Graduate School of Medicine, The University of Tokyo, Tokyo 113-0033, Japan; 4 Department of Social and Preventive Epidemiology, School of Public Health, The University of Tokyo, Tokyo 113-0033, Japan; stssasak@m.u-tokyo.ac.jp; 5 Department of Biostatistics, School of Public Health, Graduate School of Medicine, The University of Tokyo, Tokyo 113-0033, Japan; hagiwara@epistat.m.u-tokyo.ac.jp

**Keywords:** neonatal outcomes, pregnancy cohort, maternal nutrition, early life exposures

## Abstract

Perinatal cohort studies with a prospective longitudinal design are critical for determining the effects of early-life exposures on offspring’s health outcomes. The Sri Lanka Mother and Newborn Growth cohort study aims to investigate the impact of maternal nutritional and psychosocial factors on newborns’ birth weight in the Sri Lankan context. This paper presents the methodology of participant recruitment, follow-ups, an overview of measurements, and planned data analyses. This study included a nationally representative sample of Sri Lankan pregnant women recruited in their first trimester of pregnancy. Follow-up assessments were conducted once during the second and third trimesters of pregnancy and after the baby’s birth, prospectively tracking the women’s dietary intake, mental health, hemoglobin concentrations, and gestational weight gain data. Once the participants delivered their babies, the data on gestational age, sex of the newborn, birth weight, length and occipitofrontal circumference at birth, and mode of delivery were collected. Between August 2022 and August 2023, we recruited 2000 first-trimester pregnant women to the cohort and continued to follow up with them until the baby’s birth. The response rates were 90.4%, 81.4%, and 75.2% in the first, second, and third follow-ups. We plan to analyze the data in July 2024. We expect this study to provide valuable insights into various early-life exposures affecting neonatal birth weight. The study’s findings will serve as a valuable information resource for a broader scientific community, enabling the development of effective policies to prevent low-birth-weight deliveries in low-resource settings.

## 1. Introduction

David Barker, whose epidemiological studies in the United Kingdom linked an unfavorable in utero environment to postnatal metabolic diseases, proposed the "Developmental Origins of Health and Disease" (DOHaD) hypothesis in 1986 [1]. The intrauterine period is crucial for long-term health because it is a time of enormous cellular growth and differentiation. A recent review of the DOHaD hypothesis states that poor nutrition or exposure to endocrine disruptors or toxicants in utero can slow down growth and cause metabolic problems that are harmful to adult health due to changes in fetal programming [2]. Several famine studies support Baker’s hypothesis, demonstrating associations between poor intrauterine nutrition and lower glucose tolerance, increased risk of hypertension [3,4], obesity [5,6,7], and type 2 diabetes [8]. Thus, the preconception and pregnancy periods are critical windows that affect one’s lifetime health.

Maternal, infant, and child health and family planning are critical components of the current maternal healthcare system in Sri Lanka. The national maternal healthcare system provides free maternal care to all pregnant women during the antenatal, intranatal, and postnatal periods [9]. Sri Lanka has consistently improved its maternal mortality ratio (MMR) and infant mortality rate (IMR) since 1940. The MMR was 30.2 per 100,000 live births, and the IMR was 8.4 per 1000 live births in 2020 [10]. Despite sustained declines in maternal and infant mortality, Sri Lanka continues to face critical health issues affecting pregnant women, infants, and children. Among them, a high prevalence of low birth weight (LBW) is a major health concern. For many years, Sri Lanka’s LBW rate has fluctuated around 16% [10]. Given the DOHaD hypothesis and Sri Lanka’s current LBW trend, it is reasonable to assume that the country will face a significant healthcare and socioeconomic burden in the future. This burden is likely to arise due to the increased prevalence of non-communicable diseases resulting from the high number of infants with LBW, in addition to other lifestyle and environmental factors. 

A review by Abu-Saad and Fraser concluded that maternal nutrition, particularly intrauterine growth restriction, preterm birth, and LBW, is a critical factor in regulating fetal growth and birth outcomes [11]. Furthermore, it is crucial to take into account the impact of additional maternal factors, such as socioeconomic factors and other environmental exposures like active and passive smoking [2], on the fetal development and health of the offspring simultaneously. Women’s socioeconomic status might indirectly affect their newborns’ birth outcomes by influencing their diet quality, especially in developing countries [11].

There are several birth cohort studies all over the world, such as the Danish National Birth Cohort (DNBC) [12], the Norwegian Mother and Child Cohort (MoBa) [13], the BABY1000-Sydney, Australia [14], and the Northern Finland Birth Cohort 1966 (NFBC1966) [15]. High-income countries establish most of these birth cohorts, limiting the application of findings to developing nations. It is well-known that factors affecting LBW differ from country to country, even from region to region of the same country. Studies conducted in Sri Lanka suggest that maternal nutrition [16], exposure to passive smoking [17], and psychosocial well-being during pregnancy [18] are important determinants of the birth weight of newborns in the Sri Lankan context. Notwithstanding the extensive body of research demonstrating the impact of maternal nutrition on offspring’s growth and health in adulthood, the retrospective nature and small sample sizes of most of these studies have certain limitations. Therefore, prospective cohort studies are the best way to examine exposures before and during pregnancy and how they affect the offspring’s health. Additionally, they offer prospective follow-up data that aids in understanding the relationship between exposures and outcomes.

**Figure 1:**
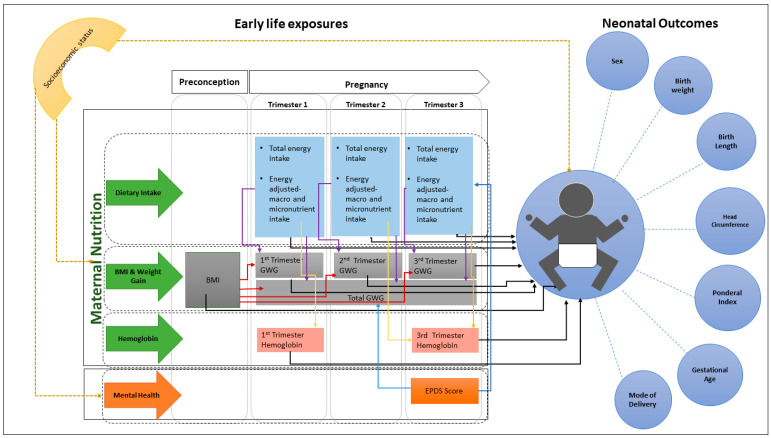
Conceptual framework of the study. BMI = body mass index; EPDS Edinburgh Postnatal Depression Scale; GWG = gestational weight gain. Ponderal index is a measure of the relationship between birth weight and body length calculated by weight (g) × 100/length (cm^3^) [19].

To date, no mother and newborn cohort studies in Sri Lanka have represented a nationwide sample of pregnant women. Thus, in 2022, we established the S-MaNGro cohort study as a nationwide prospective study to investigate the impact of maternal lifestyle factors on neonatal birth weight, with a particular emphasis on maternal nutrition. Figure 1 shows the conceptual framework for the current cohort study.

## 2. Methods and Design

### 2.1. Study Design

This study is a prospective longitudinal cohort study, aiming to establish a nationally representative cohort of 2000 maternal and newborn pairs. In 2021, the cohort’s design phase began in the Department of Nursing, Faculty of Allied Health Sciences, University of Peradeniya, Sri Lanka, in collaboration with the Department of Midwifery and Women’s Health, Graduate School of Medicine, University of Tokyo, Japan.

### 2.2. Objectives

The primary goals of the cohort are as follows:

1. estimate the total energy intake and energy-adjusted macro- and micronutrient intakes of Sri Lankan pregnant women.
2. assess the dietary behavior and the food environment among Sri Lankan pregnant women.
3. investigate the impact of maternal nutrition, mental health, and their interactions on gestational weight gain and neonatal birth weight.

### 2.3. Study Setting

Sri Lanka is a lower-middle-income country (LMIC) located in the South Asian region with a land area of approximately 65,000 km^2^ and a population of 21 million [20]. Sri Lanka has a multiethnic society, where the majority of the population is Sinhalese (74.9%), followed by Sri Lankan Tamils (11.2%), Indian Tamils (4.2%), Sri Lankan Moors (9.3%), and others. The Sinhalese speak Sinhala, whereas the Tamils and Moors people speak Tamil [20]. The country comprises nine provinces, further subdivided into 25 districts. The Ministry of Health offers free maternal and child health services through a well-established network operating at institutional and community levels. The provision of antenatal care primarily occurs through community health care centers, which are closely connected to a network of health units. Each district has clearly defined areas for health units, which are referred to as Medical Officer of Health (MOH) areas. MOH areas primarily provide preventive and promotional healthcare services to the community [9].

### 2.4. Study Population

The study population consisted of pregnant women in their first trimester of pregnancy between August 2022 and August 2023. Pregnant women seeking antenatal care from government-sponsored antenatal clinics were included in this cohort study. Women of Sri Lankan nationality who were in the first trimester of pregnancy (<12 weeks of gestation) were eligible to participate in the study. We excluded those who reported having major chronic diseases or a history of psychiatric disorders.

### 2.5. Sample Size and Sampling

This study’s main aim is to evaluate maternal nutrition’s impact on newborns’ birth weight. We calculated the minimum sample size for the study using the sample size formula for cohort studies described by Kelsey et al., 1996 [21].

(1) n1=Zα2+Z1−β2p¯q¯r+1rp1−p22

(2) $$n_{2}=rn_{1}$$

where n_1_ = number of exposed, n_2_ = number of unexposed, Zα2 Standard normal deviation for two-tailed test (was taken as 95%), $Z_{\beta }$ = standard normal deviation for one-tailed test based on beta level (taken level of power as 80%), r = ratio of unexposed to exposed (taken as 1.0), p_1_ = proportion of exposed with the outcome of interest and q_1_ = 1 − p_1_, p_2_ = proportion of unexposed with the outcome of interest and q_2_ = 1 − p_2_.

(3) $$\bar{p}=\frac{p_{1}+{rp}_{2}}{r+1}$$

and

(4) $$\bar{q}=1-\bar{p}$$

During the calculation, we considered the relative risk (RR) of an undernourished woman delivering an LBW newborn as 1.64 [95% CI 1.38–1.94] and p_1_ = 8.2% and p_2_ = 5.5%, as reported by a previous systematic review and meta-analysis [22]. We performed the calculation using the OpenEpi sample size calculator [23]. The calculated minimum sample size was 1722. The required sample size was 1980, with an expected loss to follow-up of 15%. We therefore targeted recruiting 2000 first-trimester pregnant women within one year, starting in August 2022.

A nationally representative sample of first trimester pregnant women was recruited from all nine provinces in Sri Lanka using a multistage cluster sampling method. The primary sampling units were the antenatal clinics, which are operated in each MOH area. First, we included all 25 districts. We then randomly selected one to four MOH areas within each district, depending on the population density of each district. We used the simple random sampling technique of drawing numbers to select MOH areas from each district. If a practical issue prevented the inclusion of the chosen MOH area, we selected another one within the same district using the same simple random sampling method. The number of subjects from each district was calculated using the probability proportional to size method, considering the population residing in the particular district, based on the latest available national census data for Sri Lanka [24]. We then recruited participants from each MOH area using the convenience sampling method until we reached the required sample size for each district.

### 2.6. Participant Recruitment

The pregnant women were enrolled into the study in their first trimester of pregnancy and followed up until the baby’s delivery. We invited women to participate in the study by disseminating information through antenatal clinics operating within the selected MOH areas. Before starting the data collection, we explained the study purpose, data collection methods, data collection time, their right to withdraw from the study at any stage, and the confidentiality of collected personal information to all the participants. Trained field assistants recruited participants nationwide by visiting antenatal clinics in selected MOH areas. We conducted face-to-face and online training sessions for the field assistants before and during the study implementation, as needed. All the field assistants had a healthcare-oriented background. Before beginning the data collection, we obtained informed written consent from all the participants.

### 2.7. Baseline Recruitment Survey

During the baseline recruitment survey in the first trimester of pregnancy, we asked the consenting participants to respond to a baseline questionnaire. This questionnaire covered socio-demographic information, food behavior, and the food environment. We also assessed the women’s dietary intake using a Food Frequency Questionnaire (FFQ), which has been developed and validated for Sri Lankan adults [25,26], and a 24 h dietary recall (24 h-DR). In addition, we obtained some information directly from each woman’s pregnancy card during the interview. Nutritional assessment of pregnant women is part of routine antenatal clinic care, including checking body mass index (BMI), maternal anemia, and gestational weight gain. Public health midwives calculate each woman’s BMI based on the weight and height measured at the first antenatal clinic visit (usually around 6–8 weeks gestation). To detect maternal anemia, all pregnant women undergo routine blood hemoglobin measurements at the first antenatal clinic and 28 weeks of gestation; women with hemoglobin less than 110 g/L are considered anemic. For our study, we used the recorded BMI and hemoglobin data in each woman’s pregnancy card and considered this BMI as the pre-pregnancy BMI. During the baseline survey, we also collected personal identification and contact details (name, expected delivery date, and two working phone numbers) to facilitate future follow-up interviews. We recorded these personal data separately from the research data and stored them strictly and confidentially.

### 2.8. Follow-Up Interviews

**Figure 2:**
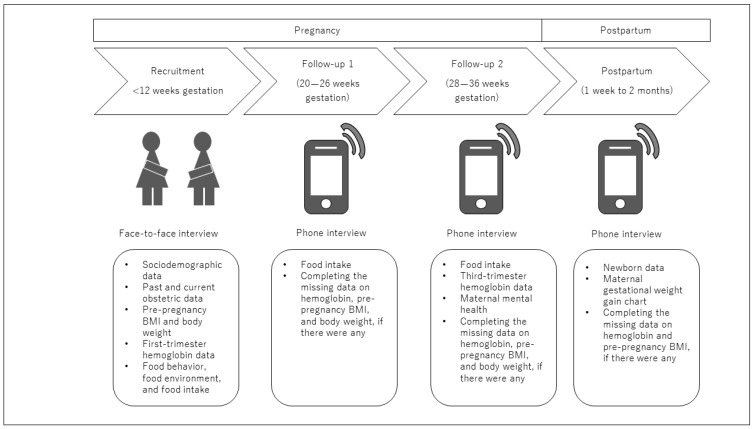
The design and process of the study.

Prospectively, we collected follow-up measurements at three distinct time points after the baseline survey through telephone interviews. Follow-up: (1) once during the second trimester (20–26 weeks of gestation); (2) once during the third trimester (28–36 weeks of gestation); and (3) once following the baby’s birth (1 week to 2 months of baby delivery). We identified the dates or periods of follow-up interviews based on each woman’s expected delivery date and contacted the subjects using the provided phone numbers. Interview dates and times were flexible to accommodate the participants’ convenience. The interviews were conducted in the participants’ native language, either Sinhala or Tamil, by the trained native language interviewers. Figure 2 summarizes the study process and includes measurements in different study phases.

### 2.9. Study Measures

Table 1 provides an overview of study measurements and data collection tools from recruitment to the final follow-up. Besides the data collection tools utilized in the baseline and the first follow-up interviews, we used the Edinburgh Postnatal Depression Scale (EPDS) [27] during the second follow-up to assess maternal mental health in the third trimester of pregnancy. Although the EPDS was initially developed and validated for postnatal use, it does not specifically address the postnatal period. Several studies revealed that the instrument has good sensitivity and specificity for detecting antenatal major depressive disorders and can be used during pregnancy to investigate maternal emotional well-being [28,29]. In the current study, interviewers used the validated versions of the EPDS in Sinhala [30] or Tamil [31], based on the participant’s native language.

### 2.10. Quality Assurance

Thirty-six field assistants were involved in participant recruitment and conducting the baseline survey, where eight interviewers conducted follow-up interviews over the phone. We provided comprehensive face-to-face and online training to all field assistants and follow-up interviewers at the outset. The principal investigator regularly updated the data collectors through WhatsApp calls, and they could always contact the principal investigator if they needed clarification during the data collection and submission process. Additionally, a specially appointed research assistant coordinated the recruitment phase of the cohort. During the recruitment phase, the principal investigator visited randomly selected data collection sites to provide supervision and feedback.

### 2.11. Data Management

The field assistants separately recorded the collected personal data (names and contact numbers) in hardcopies, keeping them locked until they submitted them to the research team. The FFQ data and the 24 h-DR data were also recorded in print materials. Once the interviews were completed, the data collectors submitted the paper-based study materials to the research team via hand delivery or registered post as sealed packets. None of the data collection papers had the participants’ personal data, like their names or telephone numbers. When the field assistants used the registered post for data submission, they separated the personal data from the research data. They posted them twice, leaving enough time for the personal data parcel to reach the destination. After receiving the personal data files from the field assistants, the research team members entered the data into an Excel sheet, creating a single personal data file that combined data from all over the country. After entering the personal data into the main personal data file, the research team assigned each subject an identification number ranging from IC-001 to IC-2000. Until the subjects received this IC-XXX number, the field assistants used a unique identification code for each participant, reflecting the district name, field assistant’s initials, and a serial number (for example, Colombo-ML-001, Colombo-ML-002, etc.).

We developed the baseline survey and EPDS questionnaires using Kobo Toolbox (https://www.kobotoolbox.org/), a software tool for field data collection developed by the Harvard Humanitarian Initiative [32]. The field assistants and the follow-up interviewers had access to the questionnaire through the KoboCollect mobile application, enabling them to enter and store data offline during the interviews. Upon concluding the interviews, the data collectors submitted the baseline survey and EPDS data to the principal investigator via the mobile application, facilitating their download in Excel format. The Kobo Toolbox encrypts the data, making it inaccessible to anyone without the private key. The principal investigator held the private password to the designed KoboToolbox account and regularly downloaded the data sent by the field assistants and follow-up interviewers at the end of each month.

The data collection papers and the personal data record papers were stored separately under lock and key in the cupboards that belonged to the principal investigator at the primary research site. Passwords protected the data entered into the Excel sheets, storing them on two hard disks exclusively used for this cohort study.

### 2.12. Modification of Nutrient Analysis Software

We modified the NutriSurvey 2007 (EBISpro, Willstaett, Germany) nutrient analysis software for Sri Lankan foods. The process of modification involved adding single food items from Sri Lankan food composition tables [33] to the NutriSurvey 2007 software. If there were any missing food items in the Sri Lankan food composition table, we used the United States Department of Agriculture (USDA) database [34]. We utilized the nutrients on food packaging labels for items like processed foods, cookies, and snacks. We used a standard recipe book to make curries and mixed dishes inside the software [35]. We adapted a procedure from a previous study to estimate portion sizes for curries and mixed dishes [36]. Modifying the nutrient analysis software for curry and mixed dishes was described earlier [16]. In brief, according to the recipes, we initially entered individual food items along with their corresponding quantities. We then selected a cooking method from the software’s available options for each recipe to estimate the weight loss resulting from various cooking techniques. The software then automatically calculates the standard macro- and micronutrient composition for 100 g of the final product. We also measured the portion sizes of each food item, such as fruits, bakery items, and biscuits (all of the average size), and adjusted the portion sizes accordingly.

After the final software modification, we checked all the relevant food items for missing values, compared them with another standard food composition table, and decided what macro- and micronutrients we would include in our analysis. When deciding which nutrients to incorporate, we prioritized the essential nutrients for fetal growth and development and those with minimal missing values in the software.

### 2.13. Data Analysis Plan

Before starting the statistical analysis, we will enter and recheck all the data in Microsoft Excel 2019. Once the analysis of FFQ and 24 h-DRs is complete, we will directly export energy, macro-, and micro-nutrient data from the modified NutriSurvey 2007 software into Microsoft Excel 2019. The statistical analysis of the data will be carried out using Minitab version 22.1. We will compare categorical variables using either the Chi-Square or Fisher’s exact test. All continuous variables will be first assessed using numerical and graphical techniques, including scatter plots, to determine if they meet the distributional assumption of the statistical tests used to analyze them. Normally distributed continuous variables will be presented as mean ± standard deviation (SD) and compared using a one-way analysis of variance (ANOVA) test. A post-hoc pairwise comparison will be performed if the null hypothesis is rejected. We will use the Pearson correlation coefficient test to examine the correlation between two continuous variables with a normal distribution and the Spearman rank correlation coefficient test for non-normal distributions. Non-normally distributed continuous variables will be expressed as median and interquartile range (IQR) and compared using Kruskal–Wallis tests. The number of miscarriages, preterm deliveries, LBW deliveries, neonatal deaths, and losses to follow-up will be presented as a percentage of the total number of participants, along with the incidence rates of outcomes of interest. EPDS data analysis will be performed as per the EPDS manual [37], and the women with a score of 9 or more will be considered at risk for depression during the third trimester of pregnancy [30,31]. We will calculate estimated total energy and energy-adjusted macro- and micronutrient values among Sri Lankan pregnant women for each trimester of the pregnancy and compare those values among different trimesters. For macronutrients (carbohydrate, protein, and fat), nutrient densities will be expressed as a proportion of energy (i.e., percentage of kcal). In contrast, micronutrients’ nutrient density will be expressed as intake (in appropriate units)/1000 kcal. The data on dietary behavior and food environment will be mostly presented as percentages and frequencies. Stepwise backward multiple binary logistic regression analysis will be carried out to evaluate the impact of maternal nutritional factors and the odds of occurring LBW after controlling for the effect of other variables (e.g., characteristics of pregnant women, income, area of residence, and confounding effects). The variance inflation factor (VIF) will be used to determine the multicollinearity, and variables with a VIF > 5 will be excluded to avoid the effects of possible multicollinearity.

R^2^ statistics and the Akaike information criterion (AIC) will be considered when selecting the final model. In addition, the Hosmer–Lemeshow criterion will be used to assess the goodness of fit for the final model. For each analysis, 95% confidence intervals will be calculated, and *p* < 0.05 will be considered statistically significant.

## 3. Results

**Figure 3:**
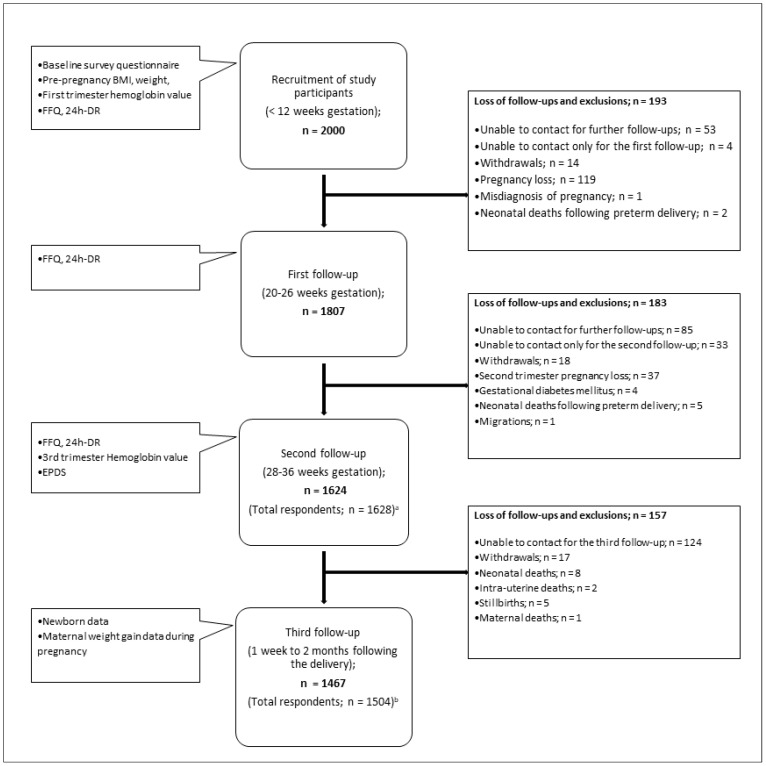
Participants recruitment and retention flow. ^a^ Women who lost contact only during the first follow-up (n = 4) but responded to the second follow-up interview. ^b^ Women who lost contact only during the first follow-up (n = 4) and only for the second follow-up (n = 33) but responded to the third follow-up interview.

For the S-MaNGro cohort study, we recruited 2000 first-trimester pregnant women, between August 2022 and August 2023. At present, the data collection process is complete. The total response rates at each stage were recorded as 90.4% (n = 1807), 81.4% (n = 1628), and 75.2% (n = 1504) in the first, second, and third follow-ups, respectively. At the final stage, we found that 73.4% (n = 1467) of participating women responded to all three follow-ups after recruitment to the study (Figure 3). Currently, we are entering the data into the modified nutrient analysis software and the Microsoft Excel 19 program. The goal is to start data analysis following data cleaning in July 2024.

## 4. Discussion

In 2012, the World Health Assembly Resolution 65.6 endorsed a comprehensive implementation plan on maternal, infant, and young children’s nutrition [38], which specified six global nutrition targets, with LBW reduction by 30% by 2025 being the third target. LBW prevalence varies significantly across different regions, with low- and middle-income countries experiencing the greatest impact. According to the available data from 2008–2012 [38], South Asian countries have to play a significant role, as they carry a disproportionate share of the global statistics of LBW. Thus, exploring all direct and indirect factors associated with LBW deliveries at the country or regional level through well-designed studies with a deeper understanding of the context is essential. Pregnancy and birth cohort studies sought to provide the most suitable approach to examine early life exposures and their possible effects on offspring.

This protocol paper presents the general approach of a nationwide prospective maternal and newborn cohort study in Sri Lanka to evaluate the impact of maternal nutritional and psychosocial factors on neonatal birth weight. To our knowledge, this is the first pregnancy cohort study in Sri Lanka that spans and focuses on nationwide representation. The cohort demonstrates satisfactory follow-up rates at the end of the data collection period.

The S-MaNGro cohort study has several strengths. The biggest strength is the sequential collection of maternal dietary data in the first trimester (<12 weeks), second trimester (20–26 weeks), and third trimester (28–36 weeks) from a nationally representative sample of pregnant women. The sequential collection of dietary data will enable us to explore the effects of maternal nutrition in different trimesters of pregnancy on birth outcomes. Secondly, the study used the multistage cluster sampling method to cover the entire nation, which includes all areas and all ethnicities in Sri Lanka. Recruitment from all 25 districts provides a more generalizable, nationally representative sample, giving more important insights into disparities by location and different ethnicities. Another distinctive feature of this cohort is its ongoing collaboration with the Department of Midwifery and Women’s Health at the University of Tokyo, the leading institution of a similar birth cohort study in Japan called the Japan Pregnancy Eating and Activity Cohort Study (J-PEACH) [39]. Given the wide disparities in funding and overall capacity for research in LMICs, it is desirable to have a meaningful and mutually beneficial global partnership with high-income countries (HICs) to facilitate LMICs’ health research needs. To address this requirement, Sustainable Development Goals’ target 17 proposes the development of inter-country partnerships between the HICs and LMICs through information sharing, technology transfer, and research opportunities [40]. The current research collaboration will allow us to compare similar types of data originating in a developed nation while obtaining intellectual, financial, and technical support for the study.

Despite its strengths, the cohort presented several challenges and lessons learned, including recruitment, follow-ups, and management issues. We sought to include participants from urban, rural, and estate areas. However, the follow-up interviews were much more challenging in rural and estate settings due to poor mobile coverage and the unavailability of personal mobiles or telephones at home. During the initial stage of recruitment, the country was experiencing a severe economic crisis, which significantly impacted families’ desire to have a child. This indirectly impacted the study, as it was hard to find the required number of first-trimester pregnant women during this critical period, which slowed down the participant recruitment process.

Overall, the findings of this maternal and newborn cohort will enable a better understanding of the effect of maternal nutrition and psychosocial factors on newborns’ growth and shed new light on modifiable risk factors for devising LBW-preventing strategies. This evidence will be critical for policymakers to make informed decisions when identifying and prioritizing interventions to address the burden and consequences of maternal malnutrition during pregnancy. In summary, this study is a new maternal and newborn cohort study that enrolled 2000 pregnant women from August 2022 to August 2023. The cohort’s data will provide valuable information that will help identify factors associated with LBW in Sri Lanka and countries with similar sociocultural and economic backgrounds.

## Figures and Tables

**Table 1:** Summary of the measures used in the baseline and follow-up surveys of the S-MaNGro cohort study.

Measurement		Baseline Survey (First Trimester)	Follow-Up 1 (Second Trimester)	Follow-Up 2 (Third Trimester)	Follow-Up 3 (Postnatal)	Method/Tool
Socio-demographics						
	Maternal data: age, civil status, ethnicity, employability, educational level, household income, area of residence	✔				Structured interviewer-administered questionnaire
Paternal data						
	Educational level, height, and weight	✔				Structured interviewer-administered questionnaire (self-reported data by the pregnant women)
Past obstetric history						
	History of miscarriages/abortions and LBW deliveries	✔				Pregnancy card-A ^a^
Details of the current pregnancy						
	Pre-pregnancy BMI, gravidity, and parity	✔				Pregnancy card-A ^a^
Biomarkers						
	Maternal hemoglobin level ^b^	✔		✔		Pregnancy card-A ^a^ (for the third-trimester data, we asked the women to tell as it appeared in the pregnancy card)
Dietary behavior						
	Food choices: specific dietary restrictions, the average monthly family income spent on food, special food preparation methods or dietary preferences, and food allergies	✔				Structured interviewer-administered questionnaire
	Eating behavior: frequency of main meals and snacks per day, average eating time for a main meal, habits of meal skipping	✔				Structured interviewer-administered questionnaire
Food environment						
	Distance from house to vegetable and fish market, mode of transportation for shopping, access to a variety of fruits and vegetables, access to good quality fruits and vegetables, having a home vegetable or fruit garden	✔				Structured interviewer-administered questionnaire
	Meal preparation during pregnancy: family support and prefer to cook or not	✔				Structured interviewer-administered questionnaire
Food and nutrition intake						
	Data on usual meat, fish, fruit, dairy products, whole grain carbohydrates, packed snacks, strong black tea, and coffee intake	✔				Structured interviewer-administered questionnaire
	Consumption of recommended folic acid supplements during pregnancy	✔				Structured interviewer-administered questionnaire
	FFQ [25,26]	✔	✔	✔		An FFQ [25,26] was used to collect information on women’s usual dietary intake in each trimester. The FFQ consists of 85 plus food items and 12 photographs. “Thriposha”, a nutrient supplement provided to all pregnant women through the national healthcare system, was included in the FFQ under the pulse group.
	24 h-DR	✔	✔	✔		A 24 h-DR was used to assess each pregnant woman’s food and beverage consumption during the previous 24 h.
Gestational weight gain						
	Body weight measurements	✔			✔	Pregnancy card-A ^a^ (for the body weight data after the recruitment, we asked the women to tell as they appeared in the pregnancy card, with the relevant gestational ages).
	Perception of pregnancy weight gain and target weight gain	✔				Structured interviewer-administered questionnaire.
Maternal mental health						
	EPDS ^c^ [27]			✔		The validated version of the EPDS was used in the participants’ native language either in Sinhala [30] or in Tamil [31].
Newborn data						
	Gestational age at delivery, sex, birth weight, length, occipitofrontal circumference at birth, and mode of delivery				✔	CHDR ^d^ (we asked the mothers to tell the newborns’ data as they appeared in the CHDR.

^a^ Every pregnant woman registered in the national maternal health care system receives two sets of maternal health records: the “A” card and the “B” card. Mothers hand-hold the “A” card, while the B card is with the relevant area public health midwife, ^b^ The Sri Lankan national maternal care system provides free hemoglobin testing to all pregnant women at the booking visit (first antenatal clinic visit) and at 28 weeks of gestation [9], ^c^ In Sri Lanka, EPDS is in a routine clinic used for screening maternal postpartum depression, but for the study purposes, we gather EPDS data during the third trimester of pregnancy, ^d^ All babies born in Sri Lanka receive a CHDR to record immunization records and growth and development data. LBW = low birth weight; BMI = body mass index; FFQ = food frequency questionnaire; 24 h-DR = 24 h dietary recall; EPDS = Edinburgh Postnatal Depression Scale; CHDR = child health developmental record.

## Data Availability

No new data were created or analyzed in this study. Data sharing is not applicable to this article.
